# Supplementary material for: Dysbiosis of Gut Microbiota and Intestinal Barrier Dysfunction in Pigs with Pulmonary Inflammation Induced by Mycoplasma hyorhinis Infection
Source: mSystems. 2022 Jun 14;7(4):e00282-22. doi: 10.1128/msystems.00282-22 (PMC9426446; doi:10.1128/msystems.00282-22)
Supplement: TABLE S1 [file msystems.00282-22-s0001.docx]

**TABLE S1** Composition and nutrient level of basal diet

| Composition | g/kg | Nutrient level | % |
| --- | --- | --- | --- |
| Maize | 720 | Digestible energy (MJ/kg) | 13.91 |
| Soybean meal | 200 | Crude protein | 15.81 |
| Wheat bran | 40 | Calcium | 0.88 |
| Dicalcium phosphate | 14 | Total phosphorus | 0.57 |
| Limestone | 10.5 | Lysine | 0.98 |
| Lysine-HCL | 2 | Methionine | 0.23 |
| Sodium chloride | 3.5 | Threonine | 0.58 |
| Premix^1^ | 10 | Tryptophan | 0.18 |
| Total | 1000 |  |  |

^1^The premix provided the diet per kg as the following: 100 mg iron, 100 mg zinc, 30 mg manganese, 10 mg copper, 0.3 mg selenium, 0.5 mg iodine, 2400 mg retinyl acetate, 25 mg cholecalciferol, 20 mg DL-a-tocopheryl acetate, 3.0 mg menadione sodium bisulfite, 2.0 mg thiamin mononitrate, 6.0 mg riboflavin, 3.0 mg pyridoxine hydrochloride, 30 mg cyanocobalamin,

20 mg nicotinic acid, 8 mg calcium pantothenate, 0.5 mg folic acid, 300 mg choline.
