# Supplementary material for: Dysbiosis of Gut Microbiota and Intestinal Barrier Dysfunction in Pigs with Pulmonary Inflammation Induced by Mycoplasma hyorhinis Infection
Source: mSystems. 2022 Jun 14;7(4):e00282-22. doi: 10.1128/msystems.00282-22 (PMC9426446; doi:10.1128/msystems.00282-22)
Supplement: TABLE S2 [file msystems.00282-22-s0002.docx]

**TABLE S2** Primer sequences for qPCR analysis

| Gene | GenBank number | Primer sequence (5′→3′) | Product size (bp) |
| --- | --- | --- | --- |
| *Mucin-2* | XM_013148044.1 | Forward: CTGATGCCACAACCCACACTCTG | 80 |
|  |  | Reverse: CAGGTGTAGCAGCAGCCAGTTC |  |
| *Occludin* | NM_001163647.2 | Forward: CAGGTGCACCCTCCAGATTG | 168 |
|  |  | Reverse: TATGTCGTTGCTGGGTGCAT |  |
| *Claudin-1* | NM_001244539.1 | Forward: GACCAGGTGAAGAAGATGCGGATG | 107 |
|  |  | Reverse: CGAGCCACTCTGTTGCCATACC |  |
| *ZO-1* | XM_005659811.1 | Forward:TGGCTCTTGCACTAGCTCTG | 116 |
|  |  | Reverse: TTGCCTGCAGTGGGTCATAG |  |
| *ZO-2* | XM_021070966.1 | Forward: CCCTCAGCCGTTGCCAGTAATG | 144 |
|  |  | Reverse: CTCTCCCACCTCGTCACTCTCTG |  |
| GAPDH | XM_021091114.1 | Forward: GATTCCACCCACGGCAAGTTCC | 129 |
|  |  | Reverse: AGCACCAGCATCACCCCATTTG |  |
